# Are Differences in Presentation of Early Lyme Borreliosis in Europe and North America a Consequence of a More Frequent Spirochetemia in American Patients?

**DOI:** 10.3390/jcm10071448

**Published:** 2021-04-01

**Authors:** Vera Maraspin, Petra Bogovič, Katarina Ogrinc, Tereza Rojko, Eva Ružić-Sabljić, Andrej Kastrin, Klemen Strle, Gary P. Wormser, Franc Strle

**Affiliations:** 1 Department of Infectious Diseases, University Medical Center Ljubljana, Japljeva 2, 1525 Ljubljana, Slovenia; vera.maraspin@kclj.si (V.M.); petra.bogovic@kclj.si (P.B.); katarina.ogrinc@kclj.si (K.O.); tereza.rojko@kclj.si (T.R.); 2 Institute for Microbiology and Immunology, Faculty of Medicine, University of Ljubljana, Zaloška cesta 4, 1000 Ljubljana, Slovenia; eva.ruzic-sabljic@mf.uni-lj.si; 3 Institute for Biostatistics and Medical Informatics, Faculty of Medicine, University of Ljubljana, Vrazov trg 2, 1000 Ljubljana, Slovenia; andrej.kastrin@mf.uni-lj.si; 4 Laboratory of Microbial Pathogenesis and Immunology, Division of Infectious Diseases, Wadsworth Center, New York State Department of Health, Albany, 120 New Scotland Ave, Albany, NY 12208, USA; klemen.strle@health.ny.gov; 5 Division of Infectious Diseases, New York Medical College, Valhalla, NY 10595, USA; GWormser@nymc.edu

**Keywords:** *Borrelia burgdorferi*, *Borrelia afzelii*, *Borrelia garinii*, Lyme borreliosis, isolation, blood, USA, Europe

## Abstract

To assess whether differences in presentation between US and European patients with early Lyme borreliosis are due to the lower rate of spirochetemia in Europe, we compared multiple variables for patients with erythema migrans (EM), restricting the analysis to subjects with a positive blood culture at the time of presentation: 93 US patients infected with *Borrelia burgdorferi* versus 183 European patients infected with *Borrelia afzelii* (No = 144) or *Borrelia garinii* (No = 39). Compared to spirochetemic Slovenian EM patients infected with *B. afzelii*, US patients with a positive blood culture significantly less often recalled a preceding tick bite at the site of the EM skin lesion, had a shorter duration of EM prior to diagnosis and more often had multiple EM lesions, regional lymphadenopathy, constitutional symptoms, an increased ESR value, a low blood lymphocyte count and detectable borrelia antibodies in acute and convalescent phase blood samples. Similar differences were observed when US patients were compared to Slovenian patients with *B. garinii* infection, but not all reached statistical significance. The findings are comparable to those previously reported for the corresponding skin culture positive patients and do not support the hypothesis that a higher frequency of spirochetemia at the time of presentation in US patients with EM, compared with European EM patients, is the reason for the observed differences.

## 1. Introduction

Lyme borreliosis (LB) is the most common tick-borne disease in Europe and North America. It predominantly affects the skin, nervous system and joints. The most frequent manifestation is erythema migrans (EM), a skin lesion which develops at the site of inoculation of the causative agent of LB (*Borrelia burgdorferi* sensu lato, Lyme borrelia) into the skin by the bite of an *Ixodid* tick. In North America, LB is nearly exclusively caused by *Borrelia burgdorferi* sensu stricto (hereafter referred to as *B. burgdorferi*), while in Europe it is predominantly caused by *Borrelia afzelii* and *Borrelia garinii* and the closely related species *Borrelia bavariensis* [1,2,3]. Although all of these species of Lyme borrelia cause EM, notable clinical and laboratory differences have been reported between EM patients who acquire the infection in the United States versus in Europe. These differences were first appreciated by comparing the findings of several independent case series on the clinical and laboratory features of EM in Europe and in the US, and verified by studies providing direct comparisons of the clinical features of culture confirmed cases of EM in the US and Europe. In these latter studies, the etiologic agent causing the EM skin lesion was identified based on a positive skin culture for *B. burgdorferi* in the US, and for *B. afzelii* or *B. garinii* in Europe [4,5,6,7]. Thus, the observed regional differences in certain clinical and laboratory features of patients with EM have been interpreted to be due to the different causative agents on the two continents. However, given the much higher rate of spirochetemia documented in patients with EM in the US compared to Europe [8,9,10,11], the question arises whether the observed differences in the clinical manifestations and laboratory test results are simply a reflection of the lower rate of spirochetemia associated with infection caused by European borrelial species.

In an effort to clarify this question, we evaluated multiple clinical and laboratory variables in European versus US EM patients, all of whom had a positive blood culture for borrelia at the time of presentation, with the aim to check whether earlier reported differences based on skin culture positive patients are also present in spirochetemic patients or not.

## 2. Materials and Methods

### 2.1. Selection of Patients

The data from the US patients included in the present study are derived from a previously published study on blood culture positive adult patients with EM from New York State [12]. These patients were evaluated at the Lyme Disease Diagnostic Center in Valhalla, New York, from 1997 to 2002.

Data from European patients are derived from a recent review of blood culture positive Slovenian patients >15 years old with a solitary EM and *B. afzelii* or *B. garinii* isolated from blood [13], and on unpublished data from patients with multiple EM with isolation of *B. afzelii* or *B. garinii* from blood. Patients were evaluated at the Lyme Borreliosis Outpatient Clinic at the Department of Infectious Diseases, University Medical Center Ljubljana, Slovenia, from 1995 through 2018. Patients who were blood culture positive for *B. afzelii* or *B. garinii* and who had either a solitary or multiple EM, defined by the criteria according to Stanek et al. [14], qualified for the present report. Clinical data for these patients were acquired using a structured questionnaire. The same clinical and laboratory variables were used for comparison of blood culture positive EM patients in Europe and the USA.

### 2.2. Serologic Testing

Acute and convalescent phase testing of serum specimens for borrelial antibodies was performed at baseline (at presentation) and at 1–4 weeks after initial testing in New York and at 7–10 weeks after initial testing in Slovenia. In New York, an enzyme-linked immunoassay was performed, while in Ljubljana in the period up to August 2011, an indirect immunofluorescent test (IFA) was performed, using a local isolate of *B. afzelii* as the antigen (titers ≥ 1:256 were considered positive) [15]. From September 2011 onwards for the Ljubljana patients, an indirect chemiluminescence immunoassay (LIAISON^®^, DiaSorin, Saluggia, Italy) with the antigens OspC and VlsE for detection of IgM antibodies and VlsE for IgG antibodies was utilized [16]; results were interpreted according to the manufacturer’s instructions.

### 2.3. Cultivation and Identification of Borrelia Strains

In New York, whole blood was collected in EDTA blood collection tubes. At least 9 mL of plasma was inoculated within 3 h into antibiotic-free Barbour–Stoenner–Kelly (BSK) medium (without gelatin) [17]. Cultures were incubated at 33 °C and examined by fluorescence microscopy every 2 weeks for a maximum of 12 weeks. Visualized spirochetes were identified as *B. burgdorferi* by PCR, as reported elsewhere [18].

In Ljubljana, whole blood (5 mL in the period from 1992 to 2000, 9 mL from 2001 onwards) was obtained by venipuncture into tubes containing sodium citrate. Blood was centrifuged, and 1 mL of plasma was inoculated into 7 mL modified Kelly Pettenkofer (MKP) medium and incubated at 33 °C. Cultures were examined weekly by dark-field microscopy for the presence of spirochetes for up to 12 weeks [19,20]. The species of isolates was identified using pulsed-field gel electrophoresis following MluI restriction of genomic DNA (MluI-restriction fragment length polymorphism, RFLP) or by PCR-based MseI-RFLP of the 5S–23S intergenic region [21,22].

### 2.4. Statistical Methods

Categorical variables were summarized with frequencies and percentages and 95% confidence intervals (CI), numeric variables with means and standard deviations.

The characteristics of the individual groups were compared using the Mann–Whitney U test for numerical variables and the chi-square test with Yates’ continuity correction for categorical variables. Due to multiple comparisons, a *p* value < 0.01 was interpreted as significant.

### 2.5. Ethics

Studies in Slovenia were approved by the Medical Ethics Committee of the Ministry of Health of the Republic of Slovenia (No 133/06/03, 38/05/06, 144/06/07, 35/05/09, 36/05/09, 127/06/10, 17/11/12, and 145/45/14), and for each patient written informed consent was obtained.

## 3. Results

Comparison of 93 US EM patients with a positive blood culture for *B. burgdorferi* with 183 European patients with a positive blood culture for *B. afzelii* (No = 144) or *B. garinii* (No = 39) revealed numerous statistically significant differences in clinical and laboratory findings. In contrast, no statistically significant differences were found when comparing the *B. afzelii* and *B. garinii* groups with each other, except for the maximum diameter of the EM skin lesion, which was larger for the *B. garinii* group (Table 1).

Compared to spirochetemic European EM patients infected with *B. afzelii*, US patients with a positive blood culture significantly less often recalled a preceding tick bite at the site of the EM skin lesion, had a shorter duration of the EM skin lesion prior to diagnosis but, nevertheless, had larger skin lesions, more often had multiple EM lesions, regional lymphadenopathy, and constitutional symptoms (headache, history of stiff neck, pain on neck flexion, history of chills/fever, body temperature > 38 °C at presentation). The US patients also more often experienced a Jarisch–Herxheimer reaction at the beginning of antibiotic treatment. The US patients more often had an increased ESR value and a low lymphocyte count in peripheral blood (i.e., a count < 1 × 10^9^/L) and, in addition, more often had detectable borrelia antibodies on testing of either acute or convalescent phase blood samples.

Similar differences were observed when the US patients were compared to European patients who had a positive blood culture for *B. garinii*, but several of these differences did not reach statistical significance (Table 1). In comparing patients with a positive blood culture for *B. garinii* to patients who had a positive blood culture for *B. burgdorferi*, the only variable that differed from the same comparisons with *B. afzelii* infected patients was that those infected with *B. garinii* had a larger diameter of the EM skin lesion than patients with *B. burgdorferi* isolated from blood; however, this difference was not statistically significant.

## 4. Discussion

The EM skin lesion is the earliest and most common sign of infection with Lyme borrelia. The diagnosis of EM is clinical and is based on the course and appearance of the skin lesion in persons exposed to ticks in areas endemic for LB. In the US, EM, as well as other manifestations of LB, are almost exclusively caused by *B. burgdorferi*, while in Central Europe the main causes of EM are *B. afzelii* (87–97%), followed by *B. garinii* (3–13%), and less commonly by other borrelial species, such as *B. burgdorferi*; the latter species are rare in Europe [23,24,25,26,27].

Numerous case series of patients with EM in North America or in Europe have been published. The data from these reports suggest several differences in the presentation of EM between the two continents. However, direct comparisons of the clinical and laboratory characteristics of US EM patients with *B. burgdorferi* infection and European EM patients with *B. afzelii* or *B. garinii* infection are limited to only four reports [4,5,6,7]. All four reports are based on positive borrelia culture results from skin. Each of these four reports evaluated from 19–119 patients from the Northeastern US with *B. burgdorferi* isolated from an EM skin lesion, in comparison to 37–200 patients from Central Europe (Austria, Slovenia) with a positive skin culture for *B. afzelii*, or to 116 patients from this same geographic area with a positive skin culture for *B. garinii*. These direct comparisons confirmed findings observed in the independent case series, providing further evidence that patients with EM in the US demonstrate certain clinical differences from patients with EM in Europe. The reported differences are that US patients with EM, compared with patients with EM in Europe, less often recall a preceding tick bite at the site of the EM skin lesion [4,6], have a shorter duration of the skin lesion until diagnosis [4,5,6,7], and the skin lesion less often has central clearing [4,6]. In addition, US patients with EM more frequently report fever and concomitant constitutional symptoms [4,5,6,7], more frequently have regional lymphadenopathy [4,6], and more often have multiple EM skin lesions [6]. The US patients also demonstrate a higher proportion of patients with an increased ESR [4,6], as well as a higher frequency of seropositivity to *B. burgdorferi* sensu lato at the time of diagnosis [4,6] and a higher seroconversion rate on convalescent phase serum samples [4,6]. However, no studies until now compared EM patients with a positive borrelia culture result from blood, which is indicative of disseminated infection.

With the exception of central clearing, which was not evaluated in the present study, all of the differences between US and European patients reported for borrelia skin culture positive EM were also demonstrated in the present study, which restricted the comparisons to patients with EM found to be spirochetemic on presentation. Furthermore, in the present study of spirochetemic patients several other differences were observed, including a higher proportion of US patients with a low lymphocyte count in peripheral blood on presentation, and the more frequent occurrence of the Jarisch–Herxheimer reaction at the beginning of antibiotic treatment in US compared with European patients. Neither of these variables, however, had been looked for in previous comparisons of skin culture positive patients with EM.

It should be noted that all of the statistically significant differences observed were found when comparing US patients with EM and *B. burgdorferi* isolated from blood with European patients with EM and *B. afzelii* isolated from blood. Although a comparison of US patients with European patients with *B. garinii* isolated from blood showed similar trends, there were fewer statistically significant differences, presumably due to the small sample size of the *B. garinii* infected patients. With the exception of a larger diameter of the EM skin lesion in the *B. garinii* infected group, compared with those infected with *B. afzelii*, no significant differences between the *B. afzelii* and *B. garinii* groups were found.

The main objective of the present study was to assess whether the clinical and laboratory differences previously found for patients infected by different borrelial species based on skin culture results would be recapitulated in EM patients with a positive blood culture at the time of presentation. A possible hypothesis was that differences in presentation between US and European patients with EM are not due to the different *Borrelia* species causing disease in the two locations, but instead are due largely to the differences in the frequency of spirochetemia in US and Europe. Indeed, spirochetemia has been associated with more frequent constitutional symptoms both in the US (89% in spirochetemic vs. 74% in nonspirochetemic patients with EM [12]) and in Europe (30–35% in spirochetemic vs. 19–21% in nonspirochetemic patients [13]). Moreover, the frequency of spirochetemia is substantially higher in the US patients with EM compared with European EM patients (in adult US patients with EM, Lyme borreliae can be detected in blood in 40–75% of cases, depending on the method used, vs. 1.2–7.7% in Europe) [8,9,10,11,13]. The discrepant rate of spirochetemia might imply that the previously demonstrated clinical and laboratory differences among the patient groups, who were distinguished based on skin culture results, might be explained in large part by the more frequent occurrence of spirochetemia in US compared with European patients with EM. However, this hypothesis was not confirmed in this study using data on patients with spirochetemia documented at the time of presentation. In the present study, we demonstrated that the reported differences in clinical presentation between US and European patients with EM are also observed in patients with a positive blood culture, and the magnitude of these differences was similar to what was reported previously based on cases identified by a positive skin culture. Thus, differences in the ability of the strains to disseminate hematogenously are probably not the main explanation for the observed clinical differences between EM caused by *B. burgdorferi* infection in the US compared with EM caused by *B. afzelli* or *B. garinii* in Europe. Other factors, such as differences in the immunogenicity, or other biologic features of these strains impacting pathogenicity, may be a greater contributing factor to the observed clinical differences.

The present study has several potentially important limitations. The analysis in the current study is based in part on previously published US data [12]; there was no Slovenian counterpart for all of the US information, and similarly, for some of the available European information there was no matching US data. Consequently, some clinical and laboratory findings of interest, such as the rate of EM skin lesion spread, the surface area of EM at presentation, the frequency of local symptoms at the site of the skin lesion, and certain laboratory test results, such as the levels of bilirubin, gamma glutamyl transferase, lactate dehydrogenase and creatine kinase levels, were not available for comparison. In addition, there were several methodologic differences. For example, the tests used for borrelia antibody evaluation in the US and Slovenia were not the same, and the convalescent phase serum samples were obtained at different time points (in the US at 1–4 weeks, in Europe at approximately 8 weeks after the first visit). Furthermore, the diagnostic approaches to determine spirochetemia were not identical. Of particular importance is that neither the level of spirochetemia, nor the duration of spirochetemia, was determined. For example, since we did not ascertain the pathogen loads in blood, we cannot exclude the possibility that the differences found in the present study were the result of higher numbers of bacterial cells in the blood of the US patients versus the European cases. In fact, no information on the borrelia burden in blood exists for European patients with LB, including EM; however, a US study revealed that the quantity of cell-associated borrelial DNA in the blood of patients with EM did not significantly correlate with the clinical, demographic or laboratory characteristics of patients with EM [28]. An indirect way to assess borrelia burden would be to compare data on the time between inoculation of the blood specimen into the culture media and the detection of growth in the US versus in Slovenia, but unfortunately this information was not available. Additionally, of potential relevance, the proportion of patients with multiple EM was higher in the spirochetemic US group than in the spirochetemic European groups, since this disparity is potentially consistent with a longer duration of spirochetemia in US cases. Finally, the findings of the present report are from the Northeastern US and Central Europe, and may, or may not, be relevant to other parts of the US or Europe, where borrelia strains with different biologic characteristics might be present.

## 5. Conclusions

At the time of presentation, US patients with EM and *B. burgdorferi* isolated from blood differ from European EM patients with *B. afzelii* or *B. garinii* isolated from blood in regard to several clinical and laboratory characteristics. Given that the diagnosis of EM is primarily clinical, awareness of these differences in disease presentation in the US and Europe has direct relevance to the care of patients. Moreover, the findings in the present study indicate that the higher frequency of spirochetemia in US patients with EM compared with EM patients in Europe is likely not the reason for the differences in clinical and laboratory features on presentation. Instead, the findings of the current study lend further support for the concept that other features of the infecting borrelia strains are the major determinants of these clinical differences between the US and Europe. These findings should stimulate efforts to determine the load of spirochetemia and to delineate the microbial genetic and biological features that impact the pathogenicity of Lyme borrelia species.

## Figures and Tables

**Table 1:** Comparison of Selected Demographic, Clinical, and Laboratory Features of Patients with Erythema Migrans Who Had a Positive Blood Culture for *Borrelia burgdorferi* (No = 93), *Borrelia afzelii* (No = 144) or *Borrelia garinii* (No = 39).

Pre-Treatment Findings	Isolation from Blood					
	United States	Slovenia		*p* Value		
	*Borrelia burgdorferi* *n* = 93	*Borrelia afzelii* *n* = 144	*Borrelia garinii* *n* = 39	*Bb* vs. *Ba*	*Bb* vs. *Bg*	*Ba* vs. *Bg*
Age (years)	48.4 ± 13	46.5 ± 16.6	49.4 ± 14.3	0.2317	0.6068	0.3237
Male sex	54 (58.1%; 47.4–68.2%)	62 (43.1%; 34.8–51.6%)	18 (46.2%; 30.1–62.8%)	0.0337	0.2881	0.8697
Tick bite ^a^	22 (23.7%; 15.5–33.6%)	95 (66.0%; 57.6–73.7%)	27 (69.2%; 52.4–83.0%)	**<0.0001**	**<0.0001**	0.8482
History of prior LB	13 (14.0%; 7.7–22.7%)	15 (10.4%; 6.0–16.5%)	4 (10.3%; 2.9–24.9%)	0.5330	0.7659	>0.9999
Duration of EM ^b^ (days)	6.9 ±5.9	12.9 ±16.7	18.7 ± 47.6	**<0.0001**	**0.0026**	0.4043
Largest diameter of EM (cm)	18.5 ± 11.4	12.9 ± 8.6	21.3 ± 12.7	**<0.0001**	**0.1034**	**<0.0001**
Multiple EM lesions	39 (41.9%; 31.8–52.6%)	28 (19.4%; 13.3–26.9%)	2 (5.1%; 0.6–17.3%)	**0.0003**	**<0.0001**	0.0576
Regional lymphadenopathy	46 (49.5%; 38.9–60.0%)	2/118 (1.7%; 0.2–6.0%)	0 (0%; 0.0–9.0%)	**0.0001**	**<0.0001**	>0.9999
Possible extracutaneous manifestations	5 (5.4%; 1.8–12.1%)	5 (3.5%; 1.1–7.9%)	0 (0%; 0–9.0%)	0.5197	0.3211	0.5864
Any constitutional symptom	83 (89.2%; 81.1–94.7%)	56 (38.9%; 30.9–47.4%)	13 (33.3%; 19.1–50.2%)	**<0.0001**	**<0.0001**	0.6536
History of stiff neck	38 (40.9%; 30.8–51.5%)	3 (2.1%; 0.4–6.0%)	1 (2.6%; 0.1–13.5%)	**<0.0001**	**<0.0001**	>0.9999
Pain on neck flexion	22 (23.7%; 15.5–33.6%)	1/118 (0.8%; 0.0–4.6%)	0 (0%; 0–9.0%)	**<0.0001**	**0.0021**	>0.9999
Headache	44 (47.3%; 36.9–57.9%)	26 (18.1%; 12.2–25.3%)	3 (7.7%; 1.6–20.9%)	**<0.0001**	**<0.0001**	0.1852
History of chills/fever	38 (40.9%; 30.8–51.5%)	13 (0.9%; 4.9–14.9%)	2 (5.1%; 0.6–17.3%)	**<0.0001**	**0.0001**	0.7417
Body temperature > 38 °C at presentation	5 (5.4%; 1.8–12.1%)	0 (0%; 0.0–2.5%)	1 (2.6%; 0.1–13.5%)	**0.0087**	0.6696	>0.9999
Jarisch–Herxheimer reaction	8 (8.6%; 3.8–16.3%)	0 (0%; 0.0–2.5%)	0 (0%; 0–9.0%)	**0.0005**	0.1043	-
Increased ESR	42/84 (50.0; 38.9–61.1%)	25/121 (20.7%; 13.8–20.0%)	12/33 (36.4%; 20.4–54.9%)	**<0.0001**	0.2604	0.1007
WBC < 4.5 × 10^9^/L	10/91 (11.0%; 5.4–19.3%)	15 (10.4%; 6.0–16.6%)	2 (5.1%; 0.6–17.3%)	0.9374	0.5089	0.5332
4.5–11	80/91 (87.9%, 7.9–9.4%)	127 (88.2%; 81.8–93.0%)	37 (94.9%; 82.7–99.4%)	0.8874	0.3420	0.5284
>11	1/91 (1.1%; 0.0–6.0%)	2 (1.4%; 0.2–4.9%)	0 (0%; 0–9.0%)	>0.9999	>0.9999	>0.9999
Lymphocyte count < 1 × 10^9^/L	26/91 (28.6%; 19.6–39.0%)	12 (8.3%; 4.4–14.1%)	3 (7.7%; 1.6–20.9%)	**<0.0001**	0.0168	>0.9999
Pts < 140 × 10^9^/L	7/87 (8.0%; 3.3–15.9%)	9 (6.2%; 2.9–11.5%)	0 (0%; 0–9.0%)	0.7999	0.0983	0.2080
Elevated AST	13/89 (14.6%; 8.0–32.7%)	17 (11.8%; 7.0–18.2%)	4 (10.3%; 2.9–24.9%)	0.6752	0.7005	>0.9999
Elevated ALT	20/89 (22.5%; 14.3–32.6%)	26 (18.1%; 12.2–25.3%)	9 (10.3%; 2.9–24.2%)	0.5134	0.5774	0.6328
Borrelia antibodies IgM and/or IgG baseline	58 (62.4%; 51.7–72.2%)	18 (12.5%; 7.6–19.0%)	10 (25.6%; 13.0–42.1%)	**<0.0001**	**0.0003**	0.0765
Convalescent	78/84 (92.9%; 85.1–97.3)	35 (24.3%; 17.6–32.2%)	12 (30.8%; 17.0–47.6%)	**<0.0001**	**<0.0001**	0.5399

Categorical variables are summarized with frequencies and percentages and 95% confidence intervals (CI), numeric variables with means and standard deviations. Since the original raw data from the US study [12] were not available (column “New York”), the aggregated continuous characteristics of each group were compared using the equation for *t*-test for unequal variances (i.e., “Bb vs. Ba” and “Bb vs. Bg” for variables Age, Duration of EM, and Largest diameter of EM). Bb: *Borrelia burgdorferi*; Ba: *Borrelia afzelii*; Bg: *Borrelia garinii*; LB: Lyme borreliosis; EM: erythema migrans; ESR: erythrocyte sedimentation rate (normal range was defined as follows: men: age ≤ 50 years, 0–15 mm/h, age > 50 years, 0–20 mm/h; women: age ≤ 50 years, 0–20 mm/h, age > 50 years, 0–30 mm/h); WBC: white blood cell (normal: 4–10 × 10^9^/L); Pts: platelets (normal: 140–340 × 10^9^/L); AST: aspartate aminotransferase (normal: US: ≤50 U/L; Slovenia: <0.58 µkat/L); ALT: alanine aminotransferase (normal: US: ≤50 U/L; Slovenia: <0.74 µkat/L). ^a^ At the site of the EM skin lesion. ^b^ At enrollment.

## Data Availability

Data are contained within the article.
